# Old World Monkeys are less than ideal transplantation models for testing pig organs lacking three carbohydrate antigens (Triple-Knockout)

**DOI:** 10.1038/s41598-020-66311-3

**Published:** 2020-06-17

**Authors:** Takayuki Yamamoto, Hayato Iwase, Diyan Patel, Abhijit Jagdale, David Ayares, Douglas Anderson, Devin E. Eckhoff, David K. C. Cooper, Hidetaka Hara

**Affiliations:** 1 Xenotransplantation Program, Department of Surgery, University of Alabama at Birmingham, Birmingham, AL USA; 2 Revivicor, Blacksburg, VA USA

**Keywords:** Immunology, Zoology, Medical research, Nephrology, Urology

## Abstract

Triple-knockout (TKO) pigs (with added protective human transgenes) are likely to be optimal sources of organs for clinical organ xenotransplantation because many humans have minimal or no natural antibody to TKO pig cells. However, Old World monkeys (OWMs) have naturally-existing antibodies directed to TKO cells. We measured anti-pig IgM/IgG binding, and complement-dependent cytotoxicity to wild-type (WT), α1,3-galactosyltransferase gene-knockout (GTKO), and TKO pig peripheral blood mononuclear cells (PBMCs) using sera from humans, several OWMs, and two New World monkeys (NWMs). Furthermore, we compared survival of GTKO (n = 5) and TKO (n = 3) pig kidneys in baboons. OWMs had significantly greater IgM binding and cytotoxicity to TKO PBMCs than humans or NWMs. Mean anti-TKO IgM was significantly higher in OWMs and significantly lower in NWMs than in humans. Cytotoxicity of OWM sera to TKO PBMCs was significantly greater than of human serum, but there was no significant difference between human and NWM sera. The median survival of TKO pig kidneys (4 days) in baboons was significantly shorter than that of GTKO kidneys (136 days) (p < 0.05). Even though considered ideal for clinical xenotransplantation, the presence of naturally-existing antibodies to TKO pig cells in OWMs complicates the transplantation of TKO pig kidneys in OWMs.

## Introduction

Choosing the best nonhuman primate (NHP) model to perform pig-to-NHP xenotransplantation is crucial to determine the feasibility of pig organ transplantation in humans. Old World monkeys (OWMs), such as baboons^1–3^ or rhesus monkeys^4,5^, have been used as the preferred species for preclinical studies of pig kidney xenotransplantation. α1,3-galactosyltransferase gene-knockout (GTKO) pig kidney^2,3,5^ or heart^6,7^ grafts (with added protective human transgenes) have survived and functioned in OWMs for many months or even years. Three pig carbohydrate xenoantigens have now been deleted from pigs by knockout of the underlying genes^8,9^ (Table 1). When one or more of these xenoantigens is deleted, there is a significant reduction in the binding of human antibodies to pig cells^9^.

**Table 1:** Carbohydrate xenoantigens that have been deleted in genetically-engineered pigs.

Carbohydrate (Abbreviation)	Responsible enzyme	Gene-knockout pig
1. Galactose-α1,3-galactose (Gal)	α1,3-galactosyltransferase	GTKO
2. N-glycolylneuraminic acid (Neu5Gc)	Cytidine monophosphate-N-acetylneuraminic acid hydroxylase (CMAH)	CMAH-KO
3. Sd^a^	β-1,4N-acetylgalactosaminyltransferase	β4GalNT2-KO

However, the serum complement-dependent cytotoxicity (CDC) of rhesus monkeys (an OWM) is known to be higher against triple-knockout (TKO) pig cells than against double-knockout (GTKO/β4GalNT2KO) pig cells^4^. While most OWMs have naturally-existing antibodies against TKO pig red blood cells (pRBCs), many capuchin monkeys (a new World Monkey [NWM]) do not have such antibodies, or have antibodies at low levels, and thus more closely mimic humans with respect to the response to TKO cells^10^.

The aim of the present study was to investigate (i) anti-pig IgM/IgG binding, and (ii) serum CDC to wild-type (WT), GTKO, and TKO pig peripheral blood mononuclear cells (PBMCs) using sera from humans, four OWM species, and two NWM species, and (iii) to compare the survival of GTKO (with added human transgenes) pig kidneys with that of TKO (with added transgenes) pig kidneys in baboons using an anti-CD40mAb-based immunosuppressive regimen.

## Materials and Methods

### *In vitro* study

#### Sources of primate sera and PBMCs

Human. Serum was drawn from 7 human volunteers (22–44 years-old, of all ABO blood groups) after informed consent per the guidelines of the Institutional Review Board (IRB) of the University of Alabama at Birmingham (UAB). This protocol was approved by IRB of UAB as #300001924. Two different lot numbers of pooled human serum (pooled from 50–150 donors) were purchased from Innovative Research, Novi, MI, USA. The serum was stored at −80 °C. When required, decomplementation was carried out by heat-inactivation for 30 min at 56 °C. PBMCs were also isolated from two human volunteers (blood type A and B, respectively), as previously described^11^.

NHPs. Sera were obtained from (i) OWMs (baboons, aged 3–4 years [Michale E Keeling Center, MD Anderson Cancer Center, Bastrop, TX]; rhesus monkeys, aged 2–4 years and cynomolgus monkeys, aged 2–5 years [Alpha Genesis, Yemassee, SC]; and African green monkeys, aged 3–14 years [Wake Forest School of Medicine, Winston Salem, NC] n = 6 of each species, of all ABO blood types; and (ii) NWMs (capuchin monkeys, aged 1–25 years [Alpha Genesis] n = 9, and squirrel monkeys, aged 5–13 years [Michale E Keeling Center] n = 6 of all ABO blood types. Sera were stored at −80 °C. When required, decomplementation was carried out by heat-inactivation for 30 min at 56 °C. PBMCs were isolated from whole blood, as previously described^11^.

### Sources of pig cells

PBMCs were obtained from (i) WT, (ii) GTKO and (iii) TKO pigs (none of which expressed any human ‘protective’ transgenes) (Revivicor, Blacksburg, VA). Finally, we obtained GTKO/β4NT2KO pig cells (without expression of any human ‘protective’ transgenes). As the volumes of all primate sera were limited, we only tested baboon serum against GTKO/β4NT2KO PBMCs. All pigs were of blood type O (nonA). Isolation of PBMCs was as described previously^11,12^.

### Detection of expression of xenoantigens on selected pig and primate cells by flow cytometry

PBMCs from pigs or primates were stained for expression of Gal (by isolectin BSI-B4), Neu5Gc (chicken anti-Neu5Gc mAb), and Sd^a^ (Dolichos biflorus agglutinin, DBA), as previously described^10^.

### Binding of serum IgM and IgG to pig PBMCs by flow cytometry

Serum antibody binding to PBMCs was measured by flow cytometry, as previously described^10,11^. Briefly, isolated PBMCs (1 × 10^5^ cells/tube) were incubated with 20 µl of serum (20% final concentration or 20 µl of phosphate-buffered saline [PBS] [control]) for 2 h at 4 °C. After incubation, the cells were washed twice in 3 ml FACS buffer and centrifuged at 700 g for 5 min. The supernatant was discarded. To prevent non-specific binding, 10 µl of 10% goat serum was added. Detection of IgM or IgG binding was performed by further incubating the serum with fluorescein isothiocyanate conjugated goat anti-human IgM (μ chain specific) (ThermoFisher Scientific, Waltham, MA) at 1:50 dilution or IgG (γ chain specific) (ThermoFisher Scientific) at 1:50 dilution for 30 min in the dark at 4 °C. The samples were washed twice and the cells resuspended with 200 µl diluted (×3) fixation buffer (Becton Dickinson, San Diego, CA).

Data acquisition was measured by LSR II flow cytometry (Becton Dickinson, San Jose, CA). The results were expressed as the relative geometric mean (rGM), calculated by dividing the geometric mean fluorescence for each sample by that of the negative control, as previously described^11,12^. The data were analyzed by FlowJo software (Treestar. Ashland, OR). Each sample was measured x3.

### Complement-dependent cytotoxicity (CDC) assay for PBMCs

PBMCs (5 × 10^4^ cells in 50 µl medium (RPMI, FBS [10%], antibiotics [1%] and HEPES [1%]) were incubated with 50 µl titrated decomplemented serum at 37 °C for 30 min. After washing with PBS, medium and rabbit complement (Sigma-Aldrich, St. Louis, MO) (final concentration 10%) were added, and incubation was carried out at 37 °C for 1 h. After washing with PBS, 0.5 µl of fluorescent-reactive dye solution (LIVE/DEAD Fixable Dead Cell Stain Kits [Invitrogen by Thermo Fisher Scientific, Eugene, OR]) was added to 1 ml of the cell suspension with PBS, and incubation was performed for 30 min in the dark at 4 °C. After washing with FACS buffer, 200 µl diluted (x3) Fixation Buffer (Becton Dickinson) was added, and incubation carried out for 20 min at 4 °C. Data acquisition, i.e., percentage of dead cells, was performed with a flow cytometer (BD LSRII, BD Biosciences).

Cytotoxicity was calculated, as follows:

$$ \% \,{\rm{cytototoxicity}}=([{\rm{A}}-{\rm{C}}]/[{\rm{B}}-{\rm{C}}])\times 100$$

where A represented the percentage of dead cells, B was the maximal percentage of dead cells (PBMCs fixed with 70% ethanol), and C was the minimal percentage of dead cells (PBMCs incubated with medium only).

CDC values at varying serum concentrations (50%, 25%, 12.5%, 6.25% and 3.125%) were calculated, and a curve was generated for each serum sample.

### *In vivo* study

#### Organ-source pigs

Five types of genetically-engineered pigs (three with nine, four with six, and one with three genetic modifications, all aimed at providing protection of the graft against the immune response)^9^ (Revivicor), weighing 16–20 kg, aged 2–3 months, and of blood group O (nonA), served as sources of kidney grafts (Table 2). Whether GTKO or TKO, all pigs expressed at least one human complement-regulatory and one coagulation-regulatory protein (Table 2). Transgene expression was confirmed in all cases by flow cytometry of aortic endothelial cells of respective pigs (pAECs) (data not shown). All pig donors were negative for porcine cytomegalovirus^13^.

**Table 2:** Pig to baboon kidney transplantation (pig genetic engineering, recipient survival, and causes of death).

Baboon Number	Donor pig genetic engineered phenotype	Survival(days)	Cause of Death
**TKO group (N = 3)**			
B3917	TKO/CD46/CD55/TBM/EPCR/CD47/HO1	1	HAR
B2416	TKO/CD46/CD55/TBM/EPCR/CD47/HO1	4	acute gastric dilatation
B1417	TKO/CD46/CD55/TBM/EPCR/CD47/HO1	61	AHXR
**GTKO group (N = 5)**			
B3115	GTKO/CD46/hvWF	4	acute gastric dilatation
B10815	GTKO/CD46/TBM/EPCR/CD47/HO1	90	AHXR
B9315	GTKO/CD46/CD55/TBM/EPCR/CD39	136	infection
B17315	GTKO/CD46/CD55/EPCR/TFPI/CD47	237	infection
B17615	GTKO/CD46/CD55/EPCR/TFPI/CD47	260	Infection

AHXR = acute humoral xenograft rejection; EPCR = endothelial protein C receptor; GTKO = α1,3-galactosyltransfearse gene-knockout; HAR = hyperacute rejection; HO1 = human heme oxygenase-1; hvWF = human von Willebrand factor; TBM = thrombomodulin; TFPI = tissue factor pathway inhibitor.

### Recipient baboons

Baboons (Papio *spp*) from the Division of Animal Resources of the University of Oklahoma Health Sciences Center, Oklahoma City, OK, or from the Michale E Keeling Center, 3–4 years-old, weighing 7–10 kg, were recipients of pig kidneys (Table 2). All animals were selected for low antibody levels to TKO (TKO group) or GTKO (GTKO group) pig cells determined in our laboratory (with a rGM of anti-nonGal IgM <5.0 and no or low anti-nonGal IgG), as previously described^1,14^.

### Genetically-engineered pig-to-baboon kidney xenotransplantation (n = 8)

Intravascular catheter placement in baboons, donor pig nephrectomy, and life-supporting pig kidney transplantation in baboons have been described previously^1,15^. The baboons were divided into two groups based on the pig kidney they received – a TKO group (n = 3) and a GTKO group (n = 5). All baboons received identical immunosuppressive therapy – induction with anti-thymocyte globulin (thymoglobulin) and anti-CD20mAb (rituximab)^1^, and maintenance with anti-CD40mAb, rapamycin, and low-dose corticosteroids^1^.

Animal care was in accordance with the Guide for the Care and Use of Laboratory Animals published by the Institute of Laboratory Animal Research (8th edition, 2010). Protocols were approved by the Institutional Animal Care and Use Committees at the University of Pittsburgh (IACUC) (#13082323) and the University of Alabama at Birmingham (#20673). The approved protocols included the approach to pain relief and animal well-being. Rejection-free survival was defined based on histopathological findings at euthanasia.

### Statistical analysis

Continuous variables were expressed as mean ± standard error of the mean (SEM). Comparisons among multiple groups were performed using a Kruskal-Wallis test for continuous variables. The receiver operating characteristic (ROC) curve was used to determine cytotoxicity using PBMC cut-off points based on both the positive data (individual human serum against WT PBMCs) and the negative data (individual human serum against autologous human PBMCs). Correlations between IgM and CDC or between IgG and CDC were analyzed by calculating a Pearson correlation coefficient. Baboon survival and rejection-free survival were estimated using the Kaplan-Meier method, and the overall differences between curves were compared using the log-rank test. A p value of <0.05 was considered statistically significant. All statistical analyses were performed using social sciences software GraphPad Prism 8 (GraphPad Software, San Diego, CA).

### Ethical approval

The protocol for withdrawal of human serum was approved by the Institutional Review Board of the University of Alabama at Birmingham (#300001924). Protocols for pig and baboon studies were approved by the Institutional Animal Care and Use Committees of the University of Pittsburgh (#13082323) and the University of Alabama at Birmingham (#20673).

## Results

### *In vitro* study

#### Expression of Gal, Neu5Gc, and Sd^a^ on pig PBMCs

PBMCs of WT pigs expressed Gal, Neu5Gc, and Sd^a^ antigens, while PBMCs of GTKO pigs did not express the Gal antigen, and those of TKO pigs did not express any of the 3 carbohydrate xenoantigens (data not shown), as previously described^10^.

### Expression of Gal, Neu5Gc, and Sd^a^ on primate PBMCs

PBMCs from humans and OWMs (baboons, rhesus, cynomolgus, African green) did not express Gal, but NWMs (capuchin, squirrel) expressed Gal on PBMCs, as previously described^10^. PBMCs from humans and capuchins did not express Neu5Gc, but those from OWMs expressed Neu5Gc^10^. Sd^a^ was not expressed on any primate PBMCs^10^ (data not shown).

### IgM and IgG binding to WT, GTKO, and TKO pig PBMCs by flow cytometry

#### IgM binding

**Figure 1:**
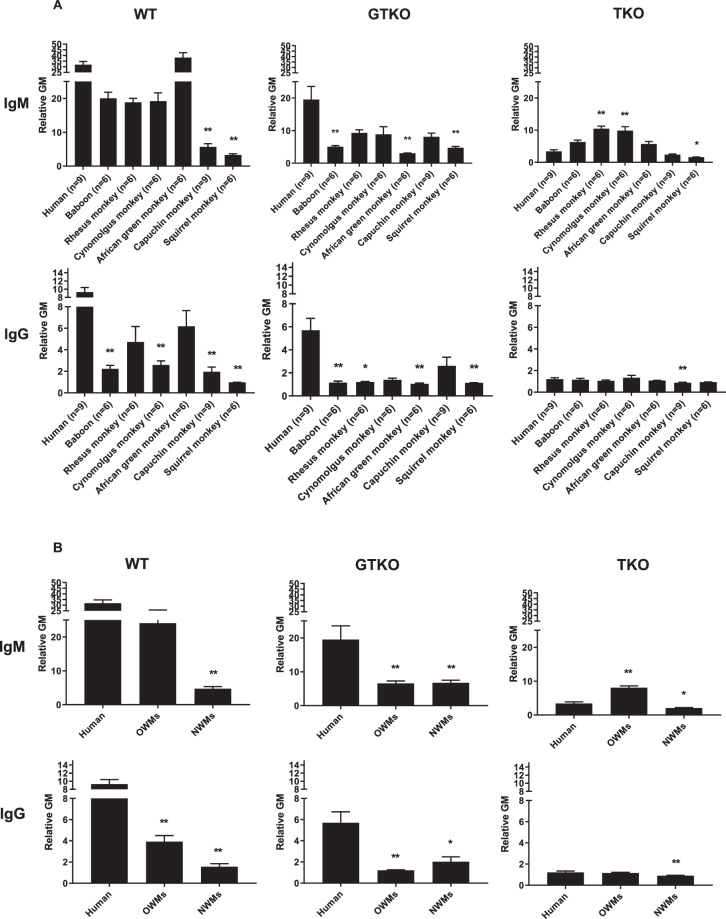
Mean levels (±SEM) of anti-WT, GTKO, and TKO pig IgM and IgG in various primate species (**A**), and summarized as human, OWM, and NWM (**B**). (**A**) Comparison of binding of preformed xenoreactive antibodies in various primate species (human [n = 9], baboon [n = 6], rhesus [n = 6], cynomolgus [n = 6], African green [n = 6], capuchin [n = 9] and squirrel monkey [n = 6]) to WT (left), GTKO (middle), and TKO (right) pig PBMCs. IgM (top) and IgG (bottom). Results are expressed as mean ± SEM. (*p < 0.05, **p < 0.01). (**B**) Comparison of binding of preformed xenoreactive antibodies in humans [n = 9], OWMs [n = 24], and NWMs [n = 15] to WT (left), GTKO (middle), and TKO (right) pig PBMCs. IgM (top) and IgG (bottom). Results are expressed as mean ± SEM. (*p < 0.05, **p < 0.01).

The mean IgM antibody binding to WT cells was significantly lower in capuchin (rGM 5.7) and squirrel (rGM 3.3) monkeys (both NWMs) than in humans (31.8) (p < 0.01) (Fig. 1A, Table 3). The mean IgM antibody binding to GTKO cells was significantly lower in baboons (rGM 5.1), African green (rGM 3.0) and squirrel (rGM 4.7) monkeys than in humans (rGM 19.5) (p < 0.01) (Fig. 1A and Table 3). In contrast, the mean IgM antibody binding to TKO cells was significantly higher in rhesus (rGM 10.5) and cynomolgus (rGM 9.8) monkeys (p < 0.01), and lower in squirrel monkeys (rGM 1.6) (p < 0.05) than in humans (rGM 3.4) (Fig. 1A and Table 3).

**Table 3:** Mean values of IgM/IgG binding and CDC among primates against 3 kinds of pig PBMCs.

	WT PBMCs			GTKO PBMCs			TKO PBMCs		
	IgM(rGM)	IgG(rGM)	CDC(%)	IgM(rGM)	IgG(rGM)	CDC(%)	IgM(rGM)	IgG(rGM)	CDC(%)
Human (n = 9)	31.8	9.3	85.7	19.5	5.7	66.7	3.4	1.2	2.7
Baboon (n = 6)	20.0	2.2^**^	91.8	5.1^**^	1.2^**^	64.4	6.3	1.2	53.5^**^
Rhesus monkey (n = 6)	18.9	4.7	92.6	9.3	1.2^*^	68.5	10.5^**^	1.1	91.8^**^
Cynomolgus monkey (n = 6)	19.2	2.6^**^	96.3	8.9	1.4	69.1	9.8^**^	1.3	90.0^**^
African green monkey (n = 6)	38.0	6.2	93.6	3.0^**^	1.1^**^	25.6^**^	5.7	1.1	77.2^**^
Capuchin monkey (n = 9)	5.7^**^	2.0^**^	49.2	8.1	2.6	72.7	2.4	0.9^**^	15.0
Squirrel monkey (n = 6)	3.3^**^	1.0^**^	62.0	4.7^**^	1.1^**^	60.0	1.6^*^	0.9	25.7

CDC = complement-dependent cytotoxicity; GTKO = α1,3-galactosyltransfearse gene-knockout; PBMC = peripheral blood mononuclear cell; rGM = relative geometric mean; TKO = triple -knockout; WT = wild type. (**p < 0.01, *p < 0.05, compared to humans).

In summary, the mean IgM antibody binding to WT cells was lower in NWMs (rGM 4.7) than in humans (rGM 31.8) (p < 0.01) (Fig. 1B). The mean IgM antibody binding to GTKO cells was lower in OWMs (rGM 6.6) and NWMs (rGM 6.8) than in humans (19.5) (p < 0.01). The mean IgM antibody binding to TKO cells was higher in OWMs (rGM 8.1) than in humans (rGM 3.4) (p < 0.01), but was lower in NWMs (rGM 2.1) (p < 0.05).

#### IgG binding

The mean IgG antbody binding to WT cells was significantly lower in baboons (rGM 2.2), cynomolgus (rGM 2.6), capuchin (rGM 2.0), and squirrel (rGM 1.0) monkeys than in humans (rGM 9.3) (p < 0.01) (Fig. 1A, Table 3). The mean IgG antibody binding to GTKO cells was significantly lower in baboons (rGM 1.2), rhesus (rGM 1.2), African green (rGM 1.1), and squirrel (rGM 1.1) monkeys than in humans (rGM 5.7) (p < 0.05) (Fig. 1A, Table 3). The mean IgG antibody binding to TKO cells was significantly lower in capuchin monkeys (rGM 0.9) than in humans (rGM 1.2) (Fig. 1A, Table 3).

In summary, the mean IgG antibody binding to WT cells and to GTKO cells was lower in OWMs (rGM for WT 3.9 and for GTKO 1.2) and NWMs (rGT for WT 1.6 and for GTKO 2.0) than in humans (rGM for WT 9.3 and for GTKO 5.7) (p < 0.05) (Fig. 1B). The mean IgG antibody to TKO cells was significantly lower in NWMs monkeys (rGT 0.9) than in humans (rGM 1.2) (p < 0.01).

These results indicate that for transplantation of kidneys from TKO pigs, IgM antibody binding will be substantially greater in OWM recipients than in humans. In contrast, for kidneys from WT or GTKO pigs, IgM antibody binding will be similar or lower in OWM recipients than in humans.

### Cytotoxicity to WT, GTKO, and TKO pig PBMCs

**Figure 2:**
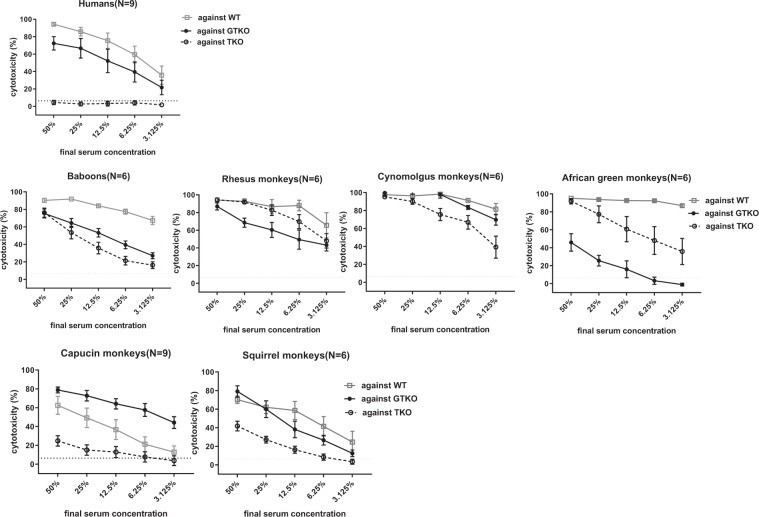
Comparison of mean serum CDC (at serum concentrations of 50% to 3.125%) of human (n = 9), baboon (n = 6), rhesus (n = 6), cynomolgus (n = 6), African green (n = 6), capuchin (n = 9), and squirrel (n = 6) monkeys against WT, GTKO, and TKO pig PBMCs. Cytotoxicity is expressed as mean ± SEM. On the y axis, the dotted line represents cut-off value of cytotoxicity (6.4%).

Cytotoxicity of <6.4% (representing the best compromise between sensitivity and specificity) was selected as the cut-off point for this assay, i.e., the lower level of detection. The outcomes of serum cytotoxicity to WT, GTKO, and TKO PBMCs, expressed in percentages, varied between the different primate species (Fig. 2).

**Figure 3:**
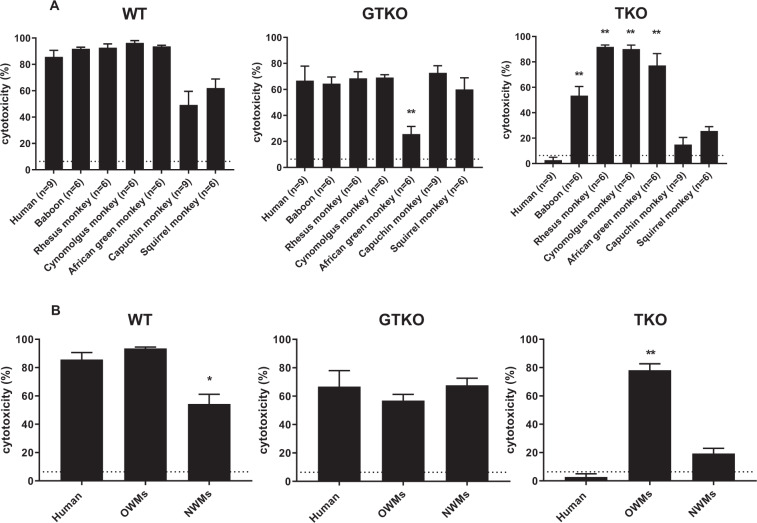
Mean (±SEM) complement-dependent cytotoxicity (CDC, at 25% serum concentration) of sera from various primate species (**A**), summarized as humans, OWMs, and NWMs (**B**) to WT, GTKO, and TKO pig PBMCs. (**A**) Comparison of mean CDC of human [n = 9], baboon [n = 6], rhesus [n = 6], cynomolgus [n = 6], African green [n = 6], capuchin [n = 9], and squirrel [n = 6]) against WT (left), GTKO (middle), and TKO (right) pig PBMCs. Results are expressed as mean ± SEM. The dotted line represents cut-off value (6.4%), below which there is no killing. (**p < 0.01). (**B**) Comparison of mean CDC of human [n = 9], OWM [n = 24], and NWM [n = 15] sera against WT (left), GTKO (middle), and TKO (right) pig PBMCs. Results are expressed as mean ± SEM. The dotted line represents cut-off value (6.4%). (*p < 0.05, **p < 0.01).

At 50% serum concentration, cytotoxicity to WT, GTKO, and TKO PBMCs among OWMs reached almost 100% (Fig. 2). Therefore, cytotoxicity at 25% serum concentration to WT, GTKO, and TKO PBMCs was used in further analysis. There was no significant difference in cytotoxicity to WT PBMCs between humans, any OWM, and any NWM (Fig. 3A, Table 3). The mean cytotoxicity in African green monkeys (26%) to GTKO PBMCs was significantly lower than in humans (67%) (p < 0.01) (Fig. 3A, Table 3). In contrast, mean cytotoxicity in baboons (53%), rhesus (92%), cynomolgus (90%) and African green (84%) monkeys to TKO PBMCs was significantly higher than in humans (2.7%) (p < 0.01) (Fig. 3A, Table 3). Cytotoxicity of NWM sera was not significantly different from that of humans.

In summary, the mean cytotoxicity in NWMs (54%) to WT PBMCs was lower than in humans (86%) (p < 0.05) (Fig. 3B). The mean cytotoxicity to GTKO PBMCs did not differ between the species (humans [67%], OWMs [58%], NWMs [68%]). Serum cytotoxicity in OWMs (74%) to TKO PBMCs was significantly higher than in humans (2.7%) (p < 0.01), but there was no significant difference between humans and NWMs (19%).

These results indicate that, for pig organ transplantation, the use of GTKO pigs will give similar results for both OWM and NWM recipient species and for human recipients. However, when TKO pigs are used, OWM species may yield a different outcome which is not representative of the condition in human recipients.

### Correlation between anti-pig IgM or IgG and CDC

To investigate the extent to which IgM or IgG antibody is associated with the results of the CDC assay, the correlation between CDC (at 25% serum concentration) and binding (rGM) of IgM or IgG antibody was evaluated.

**Figure 4:**
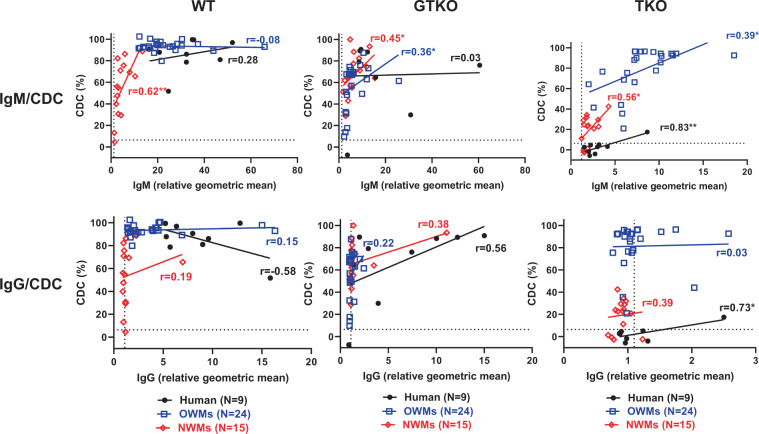
Correlation of human (n = 9, black), OWM (n = 24, blue), and NWM (n = 15, red) serum IgM (upper) and IgG (lower) antibody binding with serum complement-dependent cytotoxicity (CDC, at 25% serum concentration) to WT (left), GTKO (middle), and TKO (right) pig PBMCs. On the y axis, the dotted line represents cut-off value (6.4%) below which there is no cytotoxicity. On the x axis, the dotted line represents cut-off value (IgM:1.2, IgG:1.1) below which there is no binding. (*p < 0.05, **p < 0.01). (Note the difference in scale on the x axis between IgM and IgG. Note the difference in scale on the x axis between TKO and others).

In humans, significant correlations were found between IgM/IgG binding to TKO PBMCs and CDC (p < 0.05) (Fig. 4). In OWMs, significant correlations were found between IgM binding to both GTKO and TKO PBMCs and CDC (p < 0.05) (Fig. 4), but not for IgG binding. In NWMs, significant correlations were found between IgM binding to WT, GTKO, and TKO PBMCs and CDC (p < 0.05) (Fig. 4), but not for IgG. Even a low antibody level in OWMs might be associated with much higher CDC of TKO PBMCs than in humans (Fig. 4).

**Figure 5:**
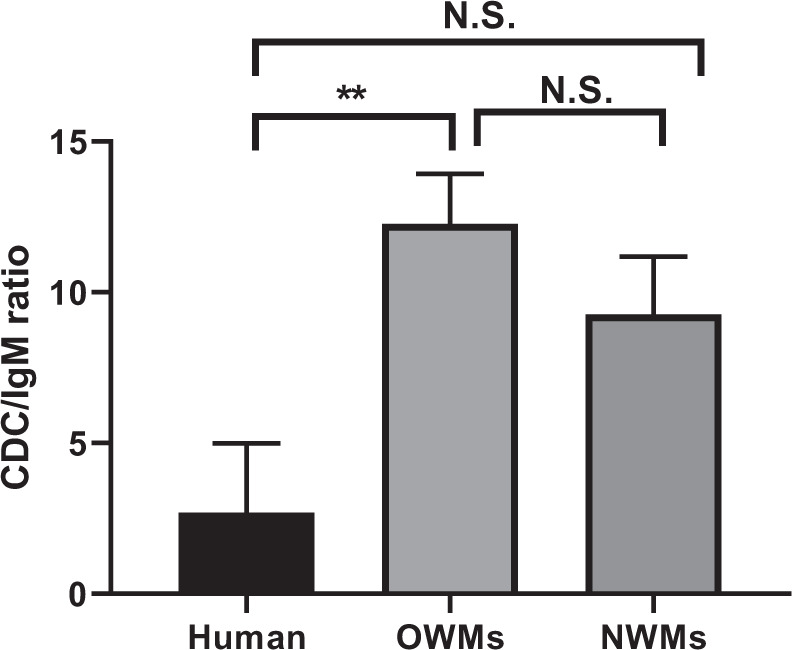
Comparison of the ratio of complement-dependent cytotoxicity (CDC, at 25% serum concentration) of TKO pig PBMCs to IgM binding in human (n = 9), OWM (n = 24), and NWM (n = 15) sera. Results are expressed as mean ± SEM. (**p < 0.01; N.S. = not significant).

Because there was significant correlation between IgM binding and CDC to TKO PBMCs in all primates, the CDC/IgM ratio (the CDC [%] divided by IgM binding [rGM]) was calculated as percentage/rGM (Fig. 5). The CDC/IgM ratio in OWMs was significantly higher than in humans (p < 0.01). In contrast, there was no significant difference between humans and NWMs.

**Figure 6:**
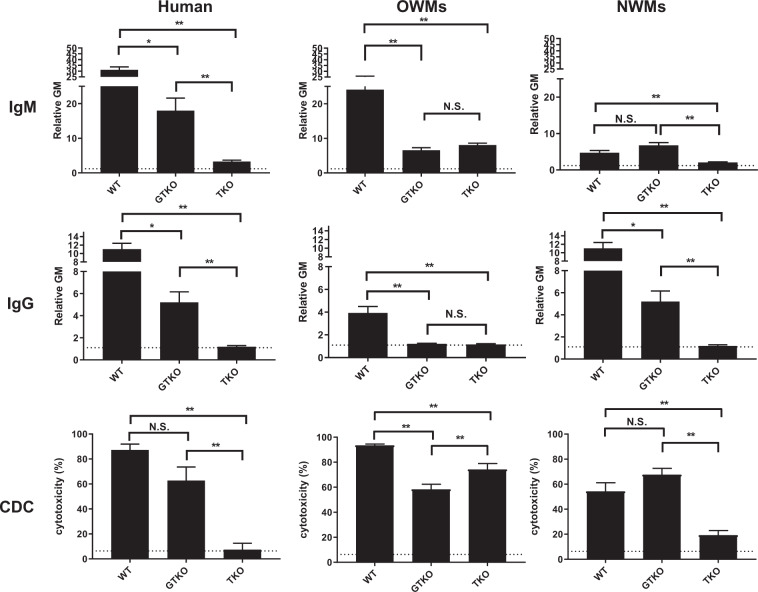
Human, OWM, and NWM IgM and IgG binding and complement-dependent cytotoxicity (CDC, at 25% serum concentration) to WT, GTKO, and TKO pig PBMCs. Results are expressed as mean ± SEM. (*p < 0.05, **p < 0.01; N.S. = not significant). On the y axis, the dotted line represents cut-off value of binding (relative GM: IgM 1.2, IgG 1.1). For CDC on the y axis, the dotted line represents cut-off value of cytotoxicity (6.4%). (Note the difference in scale on the y axis between IgM and IgG).

In summary, when one (GTKO) or more (TKO) pig xenoantigens are deleted, there is a significant reduction in the binding of human antibodies and CDC to pig PBMCs (Fig. 6). For OWMs, the mean CDC to TKO PBMCs was significantly greater than that of GTKO PBMCs, even though the levels of antibody binding were not significantly different. The binding of NWM antibodies and CDC to TKO PBMCs were lower than to GTKO PBMCs.

### *In vivo* study

#### IgM and IgG binding/cytotoxicity of recipient baboon serum to GTKO and TKO pig PBMCs

**Figure 7:**
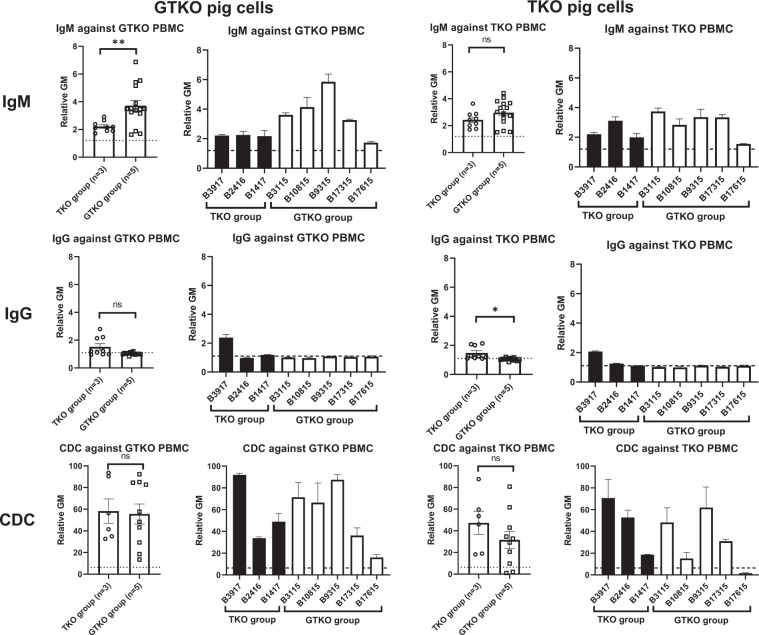
Pre-transplant IgM (upper) and IgG (middle) binding and CDC (at 25% serum concentration) (lower) of recipient baboons receiving pig kidney grafts to GTKO (left) and TKO (right) pig PBMCs. Results are expressed as mean ± SEM. On the y axis, the dotted line represents cut-off value of binding (relative GM) (IgM:1.2, IgG:1.1). For CDC, on the y axis, the dotted line represents cut-off value of cytotoxicity (6.4%). (*p < 0.05, **p < 0.01; ns = not significant).

In the baboons that received kidneys from TKO pigs (TKO group), the level of pre-transplant mean IgM antibody to TKO cells was almost 2 (rGM) (Fig. 7). In contrast, the mean rGM values of IgM antibody binding to GTKO cells in baboons in the GTKO group were between 3 and 5, except for B17615 (mean rGM:1.7), which was the longest survivor (260 days). In the TKO group, the pre-transplant CDC to TKO PBMCs was 50–70%, except for B1417 (19%), which was the longest survivor (61 days). In the GTKO group, the pre-transplant CDC to GTKO PBMCs was also 70–90%, except for B17315 (36%) and B17615 (16%), which were the 2 longest survivors (237 and 260 days).

#### Pig kidney graft survival and rejection-free survival

**Figure 8:**
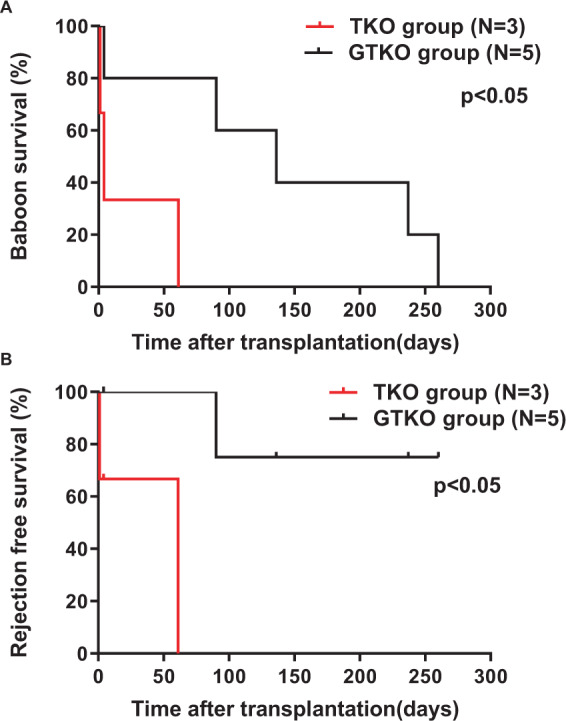
Baboon survival (**A**) and rejection-free survival (**B**) between the TKO and GTKO groups. (**A**) Baboon survival in the TKO group (red line) (N = 3) was significantly shorter than in the GTKO group (black line) (N = 5) (p < 0.05). (**B**) Rejection-free pig kidney graft survival in the TKO group was also significantly shorter than in the GTKO group (p < 0.05).

The 3 baboons in the TKO group required euthanasia on days 1, 4, and 61, respectively (Fig. 8A). The graft in B3917 (which had the highest cytotoxicity among the 3 baboons in the TKO group) failed within 24 h (and so must be considered as hyperacute rejection, confirmed by histopathology). B2416 developed hematemeses, requiring euthanasia on day 4 for acute gastric dilatation (a complication seen previously in NHPs^16^). B1417 developed acute humoral xenograft rejection on day 28; rescue therapy (high-dose steroids, anti-TNF-α antibody, intravenous immunoglobulin) extending over a month was only partially successful, and the baboon was euthanized on day 61.

The grafts in the baboons in the GTKO group functioned well for several months, except in B3115, which developed hematemeses, requiring euthanasia on day 4 for acute gastric dilatation (Table 2). As previously described^1^, in B10815 acute humoral xenograft rejection developed after withholding two doses of anti-CD40mAb (because of neutropenia), and the baboon was euthanized on day 90. The other 3 baboons required euthanasia for infectious complications on days 136, 237, and 260, but with no clinical or histopathologic signs of rejection, except possibly mild fibrosis (Table 2), as previously described^1^.

Baboon survival in the TKO group (median survival 4 days) was significantly shorter than in the GTKO group (median survival 136 days) (p < 0.05) (Fig. 8A). Rejection-free survival in the TKO group was also significantly shorter than in the GTKO group (p < 0.05) (Fig. 8B).

**Figure 9:**
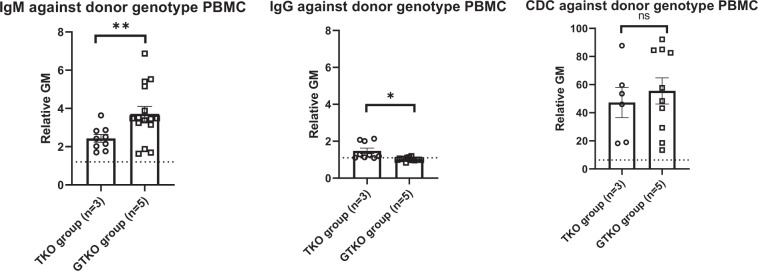
Pre-transplant IgM (left), IgG (middle) binding and CDC (at 25% serum concentration) (right) of recipient baboons receiving pig kidney grafts to either TKO pig PBMCs (TKO group) or GTKO pig PBMCs (GTKO group). Results are expressed as mean ± SEM. On the y axis, the dotted line represents cut-off value (relative GM) (IgM:1.2, IgG:1.1). For CDC, on the y axis, the dotted line represents cut-off value of cytotoxicity (6.4%). (*p < 0.05, **p < 0.01; ns = not significant).

Although the number of transplants in each group was small, the results suggest that in baboons (an OWM) the immune response to a TKO kidney graft resulting in antibody-mediated rejection is stronger than to a GTKO graft, even though the pre-transplant CDC values between the TKO and GTKO groups were not significantly different (Fig. 9).

### Additional study

#### IgM/IgG binding and CDC against GTKO, GTKO/β4GalNT2KO, and TKO pig PBMCs in baboons (n** =** 6)

**Figure 10:**
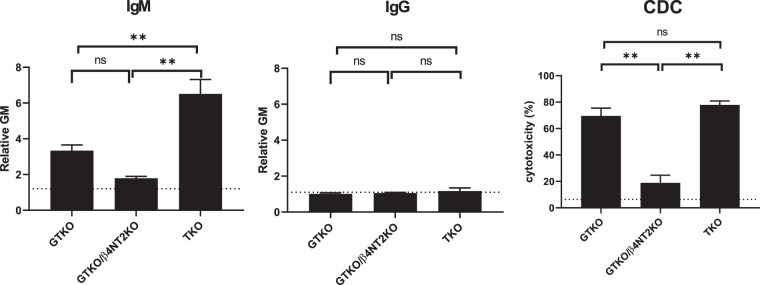
Mean levels (±SEM) of IgM/IgG binding and CDC against GTKO, GTKO/β4 NT2KO, and TKO pig PBMCs in baboon sera (n = 6). Results are expressed as mean ± SEM. On the y axis, the dotted line represents cut-off value of binding (relative GM) (IgM:1.2, IgG:1.1). For CDC, on the y axis, the dotted line represents cut-off value of cytotoxicity (6.4%). (**p < 0.01; ns = not significant).

We evaluated baboon (n = 6) serum IgM/IgG binding to, and cytotoxicity of, PBMCs from three kinds of pigs (GTKO, GTKO/β4GalNT2KO, and TKO). The mean IgM binding to GTKO/β4GalNT2KO and to GTKO cells was lower than to TKO cells (p < 0.01) (Fig. 10). Mean values of IgG binding were not significantly different. Mean percent cytotoxicity of GTKO/β4GalNT2KO cells was significantly less than to GTKO and TKO cells (p < 0.01) (Fig. 10).

## Discussion

Historically, the expression of Gal on pig vascular endothelial cells presented the most important immunobiological barrier in pig-to-primate xenotransplantation until genetically-engineered pigs were produced. For WT and GTKO pig organ transplantation, OWMs have proved to be an acceptable model with respect to translation to the condition in humans. Today, there is an increasing spectrum of pigs with genetic modifications that protect the pig tissues from the immune response (and/or correct molecular incompatibilities between pigs and primates)^9,17,18^.

There are increasing *in vitro* data indicating that TKO pig organs will prove to be a major advance over GTKO organs for transplantation into humans^9^ (Fig. 6). However, when Neu5Gc is no longer expressed in the pig (after TKO), OWMs have been found to have antibodies against an epitope in the pig, which apparently emerges upon deletion of Neu5Gc epitopes. This epitope is referred to as the “4^th^ xenoantigen”^8–10^, and most likely is a carbohydrate moiety.

We therefore compared OWM and NWM serum IgM/IgG binding to, and cytotoxicity of, WT, GTKO, and TKO PBMCs, and compared the results with those of human sera.

For OWMs, serum IgM binding and CDC to TKO PBMCs were higher than for human and NWMs (Figs. 1 and 3), and only anti-TKO IgM correlated with CDC (Fig. 4). The CDC outcome associated with anti-TKO IgM was significantly different among the 3 groups, and a main finding was that OWMs have significantly stronger cytotoxicity against TKO PBMCs than humans (Fig. 5). These data strongly suggest that antibodies against the 4^th^ xenoantigen are mainly IgM with strong cytotoxicity.

In contrast, most humans did not have natural antibodies to the 4^th^ xenoantigen, and NWMs had lower levels of IgM antibodies than OWMs. In summary, even when humans have anti-TKO antibodies, their serum cytotoxicity was much lower than that of OWMs. The cytotoxicity of NWMs serum fell between that of OWMs and humans.

Based on this vitro study, we evaluated the response to kidneys from TKO genetically-engineered pigs with six additional transgenes (which might be ideal organs for humans^9^). To our knowledge, this is the first report of kidney transplantation in baboons using TKO pigs with an additional six transgenes. Pig kidney transplantation (with an anti-CD40 mAb-based immunosuppressive regimen)^1^ was carried out in two groups of baboons that received kidneys from either TKO or GTKO pigs (each with added human transgenes) (Table 2, Figs. 7–9). The pigs in the GTKO group expressed fewer ‘protective’ transgenes than those in the TKO group, and so this might be detrimental to the outcome in the GTKO group. However, the baboons in the GTKO group showed a significantly longer kidney graft survival than those in the TKO group, and a significantly lower incidence of antibody-mediated rejection. It is tempting, therefore, to suggest that anti-TKO antibodies contributed to the rejection process.

We attempted to select the baboons on the basis of having low anti-pig antibody levels, as previously described^14^. The five baboons that received GTKO pig kidneys (GTKO group) met this criterion, but the three baboons that received a TKO pig kidney (TKO group) had anti-TKO IgG. Although the three baboons in the TKO group had lower anti-TKO IgM (compared to anti-GTKO IgM in the GTKO group), the cytotoxicity in the TKO group was similar to that in the GTKO group (Fig. 9).

Limitations of our *in vivo* study are the small number of transplants in the TKO group, and variation in donor GTKO pig genotype. Although none of the pigs that provided cells for the *in vitro* studies expressed any human transgenes, the organ-source pigs in the *in vivo* studies expressed several human transgenes. There were some differences between the GTKO and TKO pigs with respect to the presence of these additional transgenes, but these differences were not statistically significant (possibly because of the small numbers) (Supplementary Table 1*)*. It is therefore difficult to conclude that expression of any of these human transgenes influenced the transplant outcome. At least, our preliminary data indicate that the expression of various human transgenes does not affect antibody binding to the cells (Supplementary Fig. 1), which is related only to the expression of the glycan antigens.

Furthermore, the sources of kidneys were pigs all of which (except one) expressed at least one human complement-regulatory protein and all of which (except one) expressed at least one human coagulation-regulatory protein. In our experience, these are the key transgenes that protect the organ from antibody-mediated injury (in addition to lack of antigen expression). The expression of the transgenes is measured before the pigs are used as sources of kidneys, and expression is uniformly good in the great majority of cases. We therefore do not believe these variations in the organ-source pigs played a significant role in outcome.

If the baboons had not been well-immunosuppressed, it is likely that they would develop antibodies to the human proteins expressed on the pig cells. As they receive effective immunosuppressive therapy, they do not develop antibodies to pig antigens. We therefore believe it is extremely unlikely they would develop antibodies to human proteins. Although we have not specifically tested for this, we do know that, even if they become sensitized to pig antigens, they do not become sensitized to third-party allo-antigens^19^.

Our data indicate that, because of the production of antibodies to the 4^th^ xenoantigen, OWMs provide a greater barrier to survival of organs from TKO pigs than do humans or NWMs. This will make it difficult to provide the regulatory authorities with positive data to support the concept that TKO pig organs will provide long-term graft survival in humans. NWMs mimic the human immune response to TKO pig cells more closely than OWMs, but transplantation of pig organs into NWMs would be difficult because of the small size of these monkeys available in the USA (e.g., capuchin monkeys 2-4 kg, squirrel monkeys <1 kg). If a small pig kidney is transplanted into a NWM, any growth of the kidney might be problematic^2,3,20^. Furthermore, there has been much less experience of the use of immunosuppressive agents in NWMs than in OWMs, and some biological agents available today are not effective in NWMs (Yamamoto T, *et al*. manuscript submitted.).

We have previously demonstrated that baboon serum antibody binding to GTKO/β4GalNT2-KO pRBCs is significantly less than to TKO pRBCs^10^. In the present study, baboon IgM binding to, and cytotoxicity of, GTKO/β4GalNT2-KO PBMCs were significantly less than to TKO PBMCs (Fig. 10). If GTKO/β4GalNT2-KO pigs provided the organs for transplantation into OWMs, this might resolve the problem. In conclusion, when TKO pig organs are transplanted, OWMs are less than ideal as recipients as they do not mimic the human immune response.

## Supplementary information

Supplementary Information.

## Supplementary information

is available for vthis paper at 10.1038/s41598-020-66311-3.
