# Supplementary material for: Old World Monkeys are less than ideal transplantation models for testing pig organs lacking three carbohydrate antigens (Triple-Knockout)
Source: Sci Rep. 2020 Jun 17;10:9771. doi: 10.1038/s41598-020-66311-3 (PMC7300119; doi:10.1038/s41598-020-66311-3)
Supplement: Supplementary file 1 — Supplementary Information. [file 41598_2020_66311_MOESM1_ESM.docx]

**Title**: Old World monkeys are less than ideal transplantation models for testing pig organs lacking three carbohydrate antigens (Triple-Knockout)

**Authors**: Takayuki Yamamoto, MD, PhD, Hayato Iwase, MD, PhD, Diyan Patel, Abhijit Jagdale, MD, David Ayares, PhD, Douglas Anderson, MD, Devin E. Eckhoff, MD, David K.C. Cooper, MD, PhD, Hidetaka Hara, MD, PhD

*Address for correspondence:*

Hidetaka Hara MD, PhD or Takayuki Yamamoto MD, PhD

Xenotransplantation Program

University of Alabama at Birmingham (UAB)

LHRB752, 701 19^th^ Street South

Birmingham, AL 35294, USA

Tel: 205-996-7772; Fax: 205-934-8344

E-mail: [hhara@uabmc.edu](mailto:hhara@uabmc.edu) or takayukiyamamoto@uabmc.edu

***
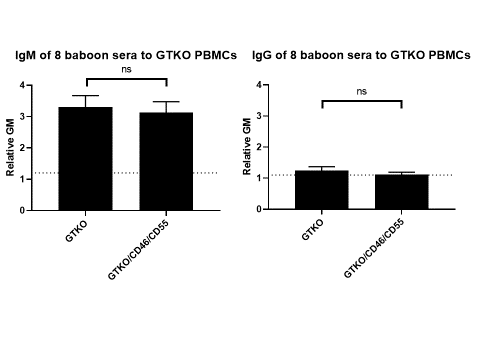
***

***Supplementary Figure 1:* Expression of human CD46 and CD55 does not influence baboon (n=8) serum antibody binding to GTKO pig PBMCs.**

Baboon serum IgM (left) and IgG (right) binding to GTKO or GTKO/CD46/CD55 pig peripheral blood mononuclear cells (PBMCs). Results are expressed as mean ± SEM. On the y axis, the dotted line represents cut-off value of binding (relative GM) (IgM:1.2, IgG:1.1), beneath which there is no significant binding. (ns = not significant).

***Supplementary Table 1:* Statistical comparison (Fisher`s exact test) of expression of human transgenes in TKO and GTKO pigs**

|  | **TKO group (N=3)** | **GTKO group (N=5)** | **P value** |
| --- | --- | --- | --- |
| **CD46** | 3/3 (100%) | 5/5 (100%) | NS |
| **CD55** | 3/3 (100%) | 3/5 (60%) | NS |
| **TBM** | 3/3 (100%) | 3/5 (60%) | NS |
| **EPCR** | 3/3 (100%) | 4/5 (80%) | NS |
| **CD47** | 3/3 (100%) | 3/5 (60%) | NS |
| **HO-1** | 3/3 (100%) | 1/5 (20%) | NS |
| **hvWF** | 0/3 (0%) | 1/5 (20%) | NS |
| **TFPI** | 0/3 (0%) | 2/5 (40%) | NS |
| **CD39** | 0/3 (0%) | 1/5 (20%) | NS |
|  | NS = not significant | | |
